# Mitochondrial Structure, Function, and Dynamics: The Common Thread across Organs, Disease, and Aging

**DOI:** 10.1155/2018/1863414

**Published:** 2018-02-08

**Authors:** Moh H. Malek, Maik Hüttemann, Icksoo Lee

**Affiliations:** ^1^Physical Therapy Program, Department of Health Care Sciences, Eugene Applebaum College of Pharmacy & Health Sciences, Wayne State University, Detroit, MI 48201, USA; ^2^Integrative Physiology of Exercise Laboratory, Department of Health Care Sciences, Eugene Applebaum College of Pharmacy & Health Sciences, Wayne State University, Detroit, MI 48201, USA; ^3^Cardiovascular Research Institute, Wayne State University School of Medicine, Detroit, MI 48201, USA; ^4^Center for Molecular Medicine and Genetics, Wayne State University School of Medicine, Detroit, MI 48201, USA; ^5^College of Medicine, Dankook University, Cheonan-si, Chungcheongnam-do 31116, Republic of Korea

Mitochondria are central to all basic and advanced cellular and organismal functions. In addition to the vast majority of cellular energy generated by these unique organelles, they are also essential signaling hubs and communicate with the rest of the cell through various means including reactive oxygen species. Their dysfunction is implicated in nearly all common diseases ranging from neuromuscular to diabetes to cancer. In this special issue, “Mitochondrial Structure, Function, and Dynamics: The Common Thread across Organs, Disease, and Aging”, we wanted to provide the readership of *Oxidative Medicine and Cellular Longevity* with a variety of examples of how mitochondria function and how different adverse conditions result in their dysfunction. The articles in this special issue can be categorized into examining mitochondria related to topics such as Alzheimer's, cancer cachexia, diabetes and wound healing, and sepsis.

In the current issue, several articles focus on the role of mitochondria in the brain. For example, P. Martín-Maestro and colleagues report a downregulation of genes involved in mitochondrial dynamics in patients diagnosed with Alzheimer's, whereas D. J. Tyrrell et al. examined blood-based bioenergetic profiles as potential biomarkers for changes in brain metabolism. D. F. Bebensee and colleagues used a transgenic murine model (Mecp2 null) to examine oxidative stress in the hippocampus region, and the potential clinical application of their findings resides in understanding a neurodevelopmental disorder called Rett syndrome. In addition, the article by K. K. Griffiths and R. J. Levy provides an excellent review on the role of mitochondrial dysfunction in autism. Taken together, these studies provide valuable insight into the role of mitochondria function in the brain.

Another tissue in which mitochondria play a critical role in bioenergetics is skeletal muscle, which comprises 50% of an individual's total body mass. Skeletal muscle is dynamic and, therefore, responds to positive stimuli such as exercise as well as negative stimuli such as aging. In this special issue, three articles are presented that provide further insight into the role of mitochondria in skeletal muscle. R. G. Feichtinger and colleagues present a case study of a child who suffered from respiratory distress syndrome soon after birth and needed to be placed on mechanical ventilation. In addition, this patient required surfactant replacement therapy. This report discusses UQCC2 mutation in neonatal encephalomyopathy with particular focus on complex III. In addition to this case report, two review articles are presented from the Hood and Carson laboratories on the “Impact of Aging and Exercise on Mitochondrial Quality Control in Skeletal Muscle” and “Disrupted Skeletal Muscle Mitochondrial Dynamics, Mitophagy, and Biogenesis during Cancer Cachexia: A Role for Inflammation”, respectively. These articles present two different scenarios (aging/exercise and cancer cachexia) and how skeletal muscle responds to these stimuli and the potential impact on mitochondrial structure and function.

Another area of research that is presented is the effect of cancer on various tissues and its role on mitochondrial function. The report by A. Koit and colleagues shows that there are differences between mitochondrial respiration as well as membrane permeability in breast cancer patients relative to patients diagnosed with colorectal cancer. The investigators use a combination of human tissue samples as well as cell culture models to distinguish differences in mitochondrial responses. In another article by R. G. Feichtinger and colleagues, the investigators explore OXPHOS complexes in two main types of gastric cancer.

Mitochondrial-targeted ROS scavengers have gained major interest in the scientific community as they go, that is, localize, to the heart of the problem, the mitochondria. Compounds, such as MitoQ, MitoTEMPO, and SKQ1, combine the ROS scavenger with the positively charged triphenylphosphonium moiety, which targets the compounds to the negatively charged inside of the mitochondria resulting in several orders of magnitude of enrichment. As such, P. Rademann and colleagues used a rodent model of sepsis to determine the effect of SkQ1 and MitoTEMPO on attenuating inflammatory responses, which did not improve animal survival and even decreased survival rates for SKQ1. Ilya and colleagues, however, used SkQ1 in a murine model of diabetes, to determine whether or not it would improve dermal wound healing. The authors reported several beneficial effects including reduced lipid peroxidation, increased *α*-smooth muscle actin-positive cells and wound closure. In conclusion, although the therapeutic benefit of mitochondrial-targeted ROS scavengers has been convincingly demonstrated for a range of acute and chronic disease conditions involving mitochondrial ROS, they may not considered a universal treatment modality with certain exceptions such as acute inflammatory signaling as seen in sepsis.

To provide more insight into the role of mitochondrial function, we provide an extensive review of cytochrome *c* oxidase subunits from function to human disease. S. Tan and E. Wong also present a review on the role and efficiency of polyphenols on mitochondrial quality control. In addition, J. D. A. Losano et al. examined the metabolic pathways in epididymal bovine sperm, whereas W. Yan and colleagues examine the interaction between testosterone and oxidative damage in orchiectomized rats.

In conclusion, all the articles presented in this special issue highlight the importance of proper mitochondrial function for healthy organ and organism performance, whereas mitochondrial dysfunction, triggered by a variety of causes ranging from specific mutations to aging as the arguably ultimate mitochondrial disease, takes center stage in an ever-increasing number of pathologies including all common diseases.

*Moh H. Malek* *Maik Hüttemann* *Icksoo Lee*

